# Biological, socio-demographic, work and lifestyle determinants of sitting in young adult women: a prospective cohort study

**DOI:** 10.1186/1479-5868-11-7

**Published:** 2014-01-24

**Authors:** Léonie Uijtdewilligen, Jos WR Twisk, Amika S Singh, Mai JM Chinapaw, Willem van Mechelen, Wendy J Brown

**Affiliations:** 1 Department of Public and Occupational Health, EMGO Institute for Health and Care Research, VU University Medical Center, Amsterdam, the Netherlands; 2 Department of Health Sciences, Section Methodology and Applied Biostatistics, VU University Medical Center, Amsterdam, the Netherlands; 3 Department of Epidemiology and Biostatistics, VU University Medical Center, Amsterdam, the Netherlands; 4 The University of Queensland, School of Human Movement Studies Brisbane, Queensland, Australia

**Keywords:** Epidemiology, Prospective studies, Sedentary lifestyle, Women’s health

## Abstract

**Background:**

Sitting is associated with health risks. Factors that influence sitting are however not well understood. The aim was to examine the biological, socio-demographic, work-related and lifestyle determinants of sitting time (including during transport, work and leisure) in young adult Australian women.

**Methods:**

Self-reported data from 11,676 participants (aged 22–27 years in 2000) in the Australian Longitudinal Study on Women’s Health were collected over 9 years in 2000, 2003, 2006 and 2009. Generalised Estimating Equations were used to examine univariable and multivariable associations of body mass index (BMI), country of birth, area of residence, education, marital status, number of children, occupational status, working hours, physical activity, smoking, alcohol intake and stress with week- and weekend-day sitting time.

**Results:**

Compared with women in the respective referent categories, (1) women with higher BMI, those born in Asia, those with less than University level education, doing white collar work, working 41–48 hours a week, current smokers, non, rare or risky/high risk drinkers and those being somewhat stressed had significantly *higher* sitting time; and (2) women living in rural and remote areas, partnered women, those with children, those without a paid job and blue collar workers, those working less than 34 hours a week, and active women had significantly *lower* sitting time.

**Conclusions:**

Among young adult Australian women, those with higher BMI, those born in Asia, those with higher level occupations and long working hours, were most at risk of higher sitting time. These results can be used to identify at-risk groups and inform intervention development.

## Introduction

Sedentary behaviours encompass a distinct class of activities, including television viewing, reading, working at a desk or computer, or driving a car [1]. These sedentary activities generally do not exceed the 1.5 metabolic equivalent (MET) intensity level, which is only slightly above the resting metabolic rate, and hence have low levels of energy expenditure [1].

Recent research shows that most adults in 20 developed and developing countries sit between 3 and 8 hours a day [2]. Driven by the current development of modern information and communication technology, electronic entertainment and motorised transport, it is expected that sitting time will increase [3]. This trend is worrying because several reviews have shown that sitting time is associated with type 2 diabetes, and CVD-related and other causes of mortality, after adjusting for physical activity [4,5]. In particular, it has been estimated that each additional hour of daily sitting is associated with a 2% increased risk in all-cause mortality. Furthermore, the association between sitting and all-cause mortality is found to be non-linear such that people with high sitting time (>7 hours a day) have even higher risk of dying (hazard ratio 5% versus 2%) [6].

Given the potential health risks of sitting, interventions to decrease sitting time are needed. However, research identifying the factors that influence sitting, and the most at risk groups, which is necessary to inform the development of such interventions, is limited [7]. Although education, age, employment status, gender, body mass index (BMI), income, smoking status, physical activity, attitudes, and depressive symptoms/quality of life have been identified as *correlates* of sitting in cross-sectional studies [8], other potential influential demographic, psychological, behavioural, social and environmental factors have not been examined yet. Moreover, there is a paucity of prospective studies examining determinants of sitting time [7,8]. The aim of this paper was therefore to investigate the biological, socio-demographic, work-related and lifestyle determinants of sitting time (including during transport, work and leisure) in young adult Australian women.

## Methods

This study was conducted as part of the Australian Longitudinal Study on Women’s Health (ALSWH), which commenced in 1996 and was designed to investigate multiple factors affecting health and well-being in three generations of women. Participants were randomly selected from the national Medicare health insurance database to represent three birth cohorts of young, mid-age and older women. The focus of this paper is on the younger cohort, born between 1973 and 1978. They were first surveyed in 1996, and then at three year intervals from 2000. In 1996, 14,792 women aged 18 to 23 years (41% response rate) completed the first mailed survey. They were broadly representative of the national female population in this age group in 1996 [9,10]. Retention rates for the following surveys were 69% (2000), 65% (2003), 68% (2006), and 62% (2009) [11]. Despite this attrition, it has been shown that biases are insufficient to preclude meaningful longitudinal analyses [12]. Further details about the rationale, recruitment procedures and protocol of the ALSWH have been reported previously [9].

As information on the main outcome measure, sitting time, was only assessed in surveys 2 to 5, we did not use survey 1 for this study. Data were included in the analyses if women provided information on both the explanatory (biological, socio-demographic, work-related or lifestyle factors) *and* outcome (sitting time) variables in the same survey, in at least one year (i.e., in 2000, 2003, 2006 or 2009). Data from women who indicated that they needed regular help with daily tasks because of a long-term illness or disability were excluded from analyses. The final sample comprised 11,676 women (79% of the baseline sample). Forty-five percent of these women completed all four surveys, while 15%, 16%, and 24% responded to one, two or three surveys, respectively.

### Measures of sitting time

Sitting time was assessed using the following question: “How many hours each day do you typically spend sitting down while doing things like visiting friends, driving, reading, watching television, or working at a desk or computer: (i) on a usual week-day and; (ii) on a usual weekend-day?”. Comparable generic sitting time questions have previously been used in the International Physical Activity Questionnaire; they showed good test-retest reliability and moderate criterion validity compared with accelerometers [13]. Assuming an average sleep time of 8 hours/day and assuming that no-one sits *all* the time, values greater than 16 hours for either week-day or weekend-day sitting were set to missing [14]. For the current analyses we used hours spent sitting on a week-day and on a weekend-day as separate outcome variables.

### Measures of potential determinants

All potential biological, socio-demographic, work-related and lifestyle determinants were assessed at every survey, except for country of birth (reported only in 1996). The only continuous and biological variable was body mass index (BMI; calculated based on self-reported body weight and body height; (weight(kg)/[height(m)]^2^). All other variables were categorized as shown in Table 1. Socio-demographic variables included country of birth, area of residence (derived from postal code), highest educational qualification, marital status, and number of children. Work-related variables included occupational status (based on the Australian Standard Classification of Occupations [15]), and hours worked per week. Lifestyle variables included physical activity (assessed by a modified version of the Active Australia questionnaire [16], and categorised as ‘inactive’ (<600 MET.min per week) or ‘active’ (≥600 MET.min per week) according to public health guidelines [17]), smoking status, and alcohol consumption (according to the (Australian) National Health and Medical Research Council (NHMRC) guidelines [18]). Stress was assessed with the Perceived Stress Questionnaire for Young Women (PSQYW) [19].

**Table 1:** Descriptive statistics presented separately for each of the four surveys

	**2000**	**2003**	**2006**	**2009**	**2000**		**2003**		**2006**		**2009**		**2000**		**2003**		**2006**		**2009**	
	**N = 9284**	**N = 8788**	**N = 8831**	**N = 7872**	**Mean (sd) sitting time hours/week-day**								**Mean (sd) sitting time hours/weekend-day**							
**Overall sitting time**					6.5	(3.3)	6.5	(3.3)	6.4	(3.4)	6.2	(3.3)	5.5	(2.8)	5.5	(2.8)	5.2	(2.7)	5.0	(2.7)
**Sitting >7 hours/day (%)**					39		41		39		36		23		23		19		17	
Age (mean (sd))	24.6 (1.5)	27.6 (1.5)	30.6 (1.5)	33.7 (1.5)																
**Biological factor**																				
BMI (mean (sd))	23.8 (4.9)	24.6 (5.4)	25.2 (5.6)	25.8 (5.8)																
**Socio-demographic factors**																				
Country of birth (%)^a^																				
Australian born*	92	92	92	93	6.5	(3.2)	6.5	(3.3)	6.4	(3.3)	6.2	(3.3)	5.5	(2.8)	5.5	(2.8)	5.2	(2.7)	5.0	(2.7)
Other English speaking country	4	4	4	4	6.6	(3.3)	6.7	(3.4)	6.5	(3.3)	6.0	(3.2)	5.6	(2.9)	5.3	(2.9)	5.3	(2.8)	4.7	(2.4)
Europe	1	1	1	1	7.1	(3.2)	7.1	(3.1)	7.3	(3.3)	6.8	(3.7)	5.1	(3.0)	5.1	(3.0)	5.8	(2.9)	4.8	(2.8)
Asia	2	2	2	2	7.9	(3.5)	8.1	(3.2)	7.8	(3.4)	7.9	(3.1)	7.0	(3.6)	6.1	(3.3)	6.3	(3.4)	5.5	(2.8)
Other	1	1	1	1	6.9	(3.3)	7.1	(3.2)	7.1	(3.4)	5.4	(3.0)	5.7	(2.5)	4.5	(2.8)	5.7	(2.9)	5.1	(3.0)
Area of residence (%)^a^																				
Urban*	55	58	60	59	6.9	(3.3)	7.1	(3.3)	6.9	(3.4)	6.6	(3.4)	5.5	(2.8)	5.5	(2.8)	5.3	(2.7)	5.0	(2.7)
Rural	41	38	35	35	6.1	(3.1)	5.9	(3.1)	5.7	(3.1)	5.6	(3.0)	5.4	(2.8)	5.5	(2.8)	5.1	(2.7)	4.8	(2.7)
Remote	4	4	4	4	5.9	(3.0)	5.7	(3.2)	5.2	(2.8)	5.5	(2.8)	5.6	(2.9)	5.7	(2.9)	5.0	(2.5)	4.8	(2.7)
Educational qualification (%)^a^																				
Less than 12 years of school	10	10	8	7	5.6	(3.1)	5.5	(3.1)	5.3	(2.9)	5.3	(3.0)	5.5	(2.8)	5.7	(2.9)	5.2	(2.8)	5.0	(2.8)
Completed 12 years of school	23	19	16	14	6.6	(3.2)	6.2	(3.2)	6.0	(3.3)	5.9	(3.2)	5.7	(2.9)	5.6	(3.0)	5.2	(2.7)	5.0	(2.8)
Post school/technical school	24	25	27	26	6.4	(3.2)	6.5	(3.3)	6.2	(3.3)	6.1	(3.3)	5.5	(2.9)	5.7	(2.9)	5.2	(2.7)	5.1	(2.9)
University degree/higher degree*	39	44	48	52	6.9	(3.2)	7.0	(3.3)	6.8	(3.4)	6.5	(3.3)	5.4	(2.7)	5.4	(2.6)	5.2	(2.7)	4.9	(2.5)
Marital status (%)^a^																				
Single*	53	35	23	17	6.8	(3.3)	7.1	(3.4)	7.3	(3.4)	7.6	(3.5)	5.7	(2.9)	5.8	(3.0)	5.8	(2.9)	5.8	(2.9)
De facto	21	20	19	15	6.7	(3.2)	6.8	(3.3)	6.7	(3.3)	6.8	(3.3)	5.5	(2.8)	5.4	(2.7)	5.4	(2.7)	5.2	(2.7)
Married	24	41	54	62	6.0	(3.2)	6.0	(3.2)	5.9	(3.2)	5.7	(3.1)	5.1	(2.6)	5.3	(2.6)	4.9	(2.5)	4.7	(2.5)
Separated/divorced/widowed	2	4	4	6	6.0	(2.9)	6.3	(3.3)	6.3	(3.3)	6.4	(3.3)	5.0	(2.9)	5.8	(3.3)	5.6	(3.1)	5.1	(2.9)
Number of children (%)^a^																				
None*	80	64	51	37	6.8	(3.3)	7.2	(3.3)	7.5	(3.3)	7.7	(3.3)	5.6	(2.8)	5.6	(2.9)	5.6	(2.8)	5.6	(2.8)
1	11	16	20	19	5.5	(2.8)	5.5	(2.9)	5.8	(3.0)	6.1	(3.0)	5.3	(2.7)	5.4	(2.8)	5.1	(2.6)	5.1	(2.7)
2	7	12	19	29	5.1	(2.7)	5.1	(2.7)	5.1	(2.9)	5.2	(2.9)	5.0	(2.7)	5.2	(2.6)	4.7	(2.4)	4.4	(2.4)
≥3	2	5	9	15	4.9	(3.0)	4.7	(2.8)	4.5	(2.6)	4.7	(2.6)	5.3	(2.8)	5.1	(2.7)	4.5	(2.5)	4.3	(2.5)
**Work-related factors**																				
Occupational status (%)^a^																				
No paid job	9	19	19	20	5.4	(2.8)	5.4	(2.8)	5.0	(2.8)	4.8	(2.7)	5.5	(2.8)	5.5	(2.7)	5.0	(2.6)	4.7	(2.5)
Blue collar	7	7	6	5	4.8	(2.8)	4.8	(2.9)	4.7	(2.8)	5.0	(3.2)	5.6	(2.8)	5.6	(2.9)	5.1	(2.9)	5.0	(2.7)
White collar	33	29	27	23	7.0	(3.3)	7.1	(3.3)	6.8	(3.4)	6.7	(3.3)	5.6	(2.9)	5.7	(3.0)	5.4	(2.8)	5.2	(2.9)
Professional*	45	44	47	49	6.7	(3.2)	7.0	(3.4)	7.0	(3.4)	6.7	(3.3)	5.4	(2.8)	5.4	(2.7)	5.3	(2.7)	5.0	(2.6)
Hours worked per week (%)^a^																				
None	15	17	18	20	5.9	(3.0)	5.5	(2.8)	5.0	(2.7)	5.0	(2.8)	5.7	(2.9)	5.5	(2.7)	5.0	(2.6)	4.8	(2.6)
1-15	10	11	11	13	6.3	(3.0)	5.7	(2.8)	4.9	(2.9)	4.7	(2.5)	5.7	(2.8)	5.3	(2.6)	4.8	(2.6)	4.5	(2.5)
16-24	8	8	10	13	6.1	(2.9)	5.7	(3.0)	5.5	(3.0)	5.3	(2.9)	5.5	(2.9)	5.4	(2.7)	4.9	(2.6)	4.6	(2.5)
25-34	9	8	9	10	5.7	(2.9)	5.8	(3.1)	5.8	(3.1)	6.1	(3.1)	5.5	(2.9)	5.3	(2.8)	5.0	(2.5)	5.0	(2.7)
35-40*	28	25	24	20	6.8	(3.2)	7.2	(3.3)	7.3	(3.2)	7.5	(3.2)	5.5	(2.8)	5.7	(3.0)	5.5	(2.7)	5.4	(2.8)
41-48	19	19	16	14	7.1	(3.4)	7.4	(3.4)	7.7	(3.3)	7.8	(3.3)	5.4	(2.7)	5.5	(2.8)	5.5	(2.8)	5.3	(2.7)
≥49	12	12	11	10	6.8	(3.5)	7.4	(3.7)	7.5	(3.7)	7.4	(3.7)	5.3	(2.9)	5.5	(2.8)	5.5	(2.9)	5.0	(2.8)
**Lifestyle factors**																				
Physical activity (%)^a^																				
Active	55	55	49	46	6.4	(3.2)	6.6	(3.3)	6.4	(3.3)	6.3	(3.3)	5.3	(2.7)	5.4	(2.7)	5.1	(2.6)	4.9	(2.6)
Inactive*	44	44	49	51	6.7	(3.3)	6.6	(3.3)	6.4	(3.4)	6.2	(3.3)	5.7	(2.9)	5.7	(2.9)	5.3	(2.8)	5.0	(2.7)
Smoking status (%)^a^																				
Non smoker*	57	57	58	60	6.6	(3.2)	6.7	(3.3)	6.5	(3.3)	6.3	(3.3)	5.4	(2.7)	5.5	(2.8)	5.2	(2.7)	4.9	(2.6)
Ex-smoker	14	19	22	26	6.3	(3.1)	6.5	(3.2)	6.2	(3.3)	6.1	(3.3)	5.3	(2.8)	5.4	(2.7)	5.1	(2.7)	4.9	(2.7)
Current smoker	28	24	19	14	6.5	(3.4)	6.3	(3.3)	6.5	(3.5)	6.2	(3.3)	5.7	(3.0)	5.7	(2.9)	5.5	(2.9)	5.2	(3.0)
Alcohol consumption (%)^a^																				
Non drinker	9	8	10	12	6.3	(3.1)	6.1	(3.4)	5.8	(3.3)	5.8	(3.2)	5.7	(2.8)	5.7	(3.0)	5.2	(2.7)	5.1	(2.9)
Rare drinker	29	27	25	24	6.3	(3.2)	6.2	(3.2)	6.0	(3.2)	6.0	(3.2)	5.6	(2.9)	5.7	(2.9)	5.3	(2.8)	5.1	(2.7)
Low risk drinker*	58	61	60	60	6.7	(3.2)	6.7	(3.3)	6.6	(3.4)	6.3	(3.3)	5.4	(2.7)	5.4	(2.7)	5.1	(2.6)	4.9	(2.6)
Risky/high risk drinker	4	4	4	4	6.7	(3.3)	7.1	(3.1)	7.0	(3.6)	6.6	(3.6)	5.9	(3.1)	6.0	(3.0)	6.0	(3.1)	5.3	(3.0)
Stress (%)^a^																				
Being not stressed*	50	54	56	51	6.2	(3.2)	6.4	(3.3)	6.2	(3.3)	6.0	(3.2)	5.3	(2.7)	5.4	(2.7)	5.0	(2.6)	4.8	(2.6)
Being somewhat stressed	50	46	44	49	6.9	(3.3)	6.8	(3.3)	6.7	(3.4)	6.5	(3.3)	5.7	(2.9)	5.7	(2.9)	5.5	(2.9)	5.1	(2.7)

BMI, body mass index; ^a^Due to rounding and missing data, not all percentages of the categorical independent variables add up to 100%.

*These categories are used as reference categories in the GEE analyses.

Copies of all the surveys are available on the ASLWH website (http://www.ALSWH.org.au).

The ALSWH was approved by the University of Queensland and the University of Newcastle Ethics Committees. All participants gave their written informed consent.

### Analyses

Descriptive statistics (mean(sd), proportions) are presented for the independent variables and dependent variables (i.e., week-day and weekend-day sitting time), for each of the four surveys separately. Univariable linear Generalized Estimating Equations (GEE) with an exchangeable correlation structure were used to assess the associations of the independent variables with week-day and weekend-day sitting time. GEE models typically capture the changing status of all the determinants and sitting time, as well as the relationships between them, over time. We additionally generated multivariable GEE models for week-day and weekend-day sitting time, including all independent variables (i.e., BMI, country of birth, area of residence, educational qualification, marital status, number of children, occupational status, hours worked per week, physical activity, smoking status, alcohol consumption and stress). For each model, independent variables with the highest *p*-values were removed stepwise until only variables with *p* < .05 remained. All univariable and multivariable analyses were adjusted for age. The distribution of week- and weekend-day sitting was checked before performing the main analyses. Statistical analyses were conducted using SPSS version 18.0.

To test the robustness of our results, univariable and multivariable GEE analyses were repeated for women who completed all four surveys (n = 5224). Results from these analyses were compared with the results of the original sample (n = 11,676).

## Results

### Sample characteristics

The outcome variables week-day and weekend-day sitting were normally distributed. Descriptive data on biological, socio-demographic factors, work-related and lifestyle-related factors and sitting time are presented in Table 1.

On average, week-day sitting time was 6.5 hours per day in 2000 and slightly declined over time to 6.2 hours per day in 2009. Weekend-day sitting time declined over time as well; from 5.5 hours per day in 2000 to 5.0 hours per day in 2009. The percentage of women sitting >7 hours per week ranged from 36 (2009) to 41 (2000) on week-days and from 17 (2009) to 23 (both 2000 and 2003) on weekend-days. In 2000, two thirds of the women had completed only school education, more than half were single, and one fifth had a child. By 2009, more than half the women had completed a University or higher degree, almost two thirds were married, and more than 60% had at least one child.

### Multivariable associations with higher sitting time

Univariable associations between potential determinants and week-day and weekend-day sitting time are presented in Table 2, and multivariable models are shown in Table 3. As there were only minor differences between the univariable and multivariable findings, only the multivariable models (Table 3) are described here.

**Table 2:** **Age-adjusted**^**a **^**univariable GEE analyses presenting associations of biological, socio-demographic, work-related and lifestyle factors with sitting**

	**Week-day sitting (hours/day)**		**Weekend-day sitting (hours/day)**	
**Explanatory variable *(Reference)***	**B (95% CI)**		**B (95% CI)**	
**Biological factor**				
BMI^b^	0.12***	(0.08; 0.16)	0.25***	(0.22; 0.29)
**Socio-demographic factors**				
Country of birth *(Australian born)*				
Other English speaking country	0.08	(−0.18; 0.34)	−0.05	(−0.25; 0.16)
Europe	0.80**	(0.29; 1.30)	−0.07	(−0.51; 0.36)
Asia	1.39***	(1.02; 1.76)	0.94***	(0.61; 1.27)
Other	0.18	(−0.37; 0.72)	0.03	(−0.43; 0.49)
Area of residence *(Urban)*				
Rural	−0.93***	(−1.01; −0.85)	−0.10**	(−0.17; −0.03)
Remote	−0.98***	(−1.17; −0.78)	−0.01	(−0.18; 0.16)
Educational qualification *(University degree/higher degree)*				
Less than 12 years of school	−1.23***	(−1.38; −1.09)	0.18**	(0.05; 0.31)
Completed 12 years of school	−0.55***	(−0.67; −0.43)	0.15**	(0.05; 0.25)
Post school/technical school	−0.51***	(−0.62; −0.40)	0.16***	(0.07; 0.25)
Marital status *(Single)*				
De facto	−0.09	(−0.19; 0.01)	−0.24***	(−0.33; −0.15)
Married	−0.81***	(−0.91; −0.72)	−0.54***	(−0.62; −0.46)
Separated/divorced/widowed	−0.51***	(−0.70; −0.32)	−0.28**	(−0.46; −0.09)
Number of children *(None)*				
1	−1.44***	(−1.53; −1.35)	−0.35***	(−0.44; −0.27)
2	−2.17***	(−2.27; −2.07)	−0.84***	(−0.93; −0.76)
≥3	−2.62***	(−2.76; −2.48)	−0.99***	(−1.11; −0.87)
**Work-related factors**				
Occupational status *(Professional)*				
No paid job	−1.50***	(−1.60; −1.40)	−0.16***	(−0.25; −0.08)
Blue collar	−1.69***	(−1.83; −1.54)	0.05	(−0.09; 0.18)
White collar	−0.05	(−0.15; 0.04)	0.08	(0.00; 0.16)
Hours worked per week *(35–40)*				
None	−1.55***	(−1.66; −1.45)	−0.18***	(−0.27; −0.09)
1-15	−1.42***	(−1.53; −1.30)	−0.31***	(−0.41; −0.21)
16-24	−1.20***	(−1.32; −1.08)	−0.32***	(−0.43; −0.22)
25-34	−0.95***	(−1.07; −0.83)	−0.21***	(−0.32; −0.10)
41-48	0.17**	(0.08; 0.27)	−0.02	(−0.11; 0.07)
≥49	0.08	(−0.05; 0.20)	−0.03	(−0.14; 0.08)
**Lifestyle factors**				
Being active	−0.07	(−0.13; 0.00)	−0.19***	(−0.25; −0.13)
Smoking status *(Non smoker)*				
Ex-smoker	−0.23***	(−0.34; −0.13)	0.00	(−0.08; 0.09)
Current smoker	−0.15**	(−0.26; −0.05)	0.20***	(0.11; 0.29)
Alcohol consumption *(Low risk drinker)*				
Non drinker	−0.54***	(−0.67; −0.41)	0.17**	(0.05; 0.28)
Rare drinker	−0.34***	(−0.42; −0.26)	0.17***	(0.10; 0.24)
Risky/high risk drinker	0.20*	(0.00; 0.39)	0.39***	(0.21; 0.56)
Being somewhat stressed	0.30***	(0.23; 0.37)	0.25***	(0.19; 0.31)

BMI, body mass index; CI, confidence interval; ^a^Women’s age at each survey (not only baseline age) was included in the model; ^b^Values for BMI signify 5 steps (i.e. 5 BMI-points) on the determinant scale; *p < .05; **p < .01; ***p < .001.

**Table 3:** **Adjusted**^**a **^**multivariable GEE analyses presenting associations of biological, socio-demographic, work-related and lifestyle factors with sitting**

	**Weekend-day sitting (hours/day)**		**Weekend-day sitting (hours/day)**	
**Explanatory variable *(Reference)***	**B (95% CI)**		**B (95% CI)**	
**Biological factor**				
BMI^b^	0.19***	(0.15; 0.23)	0.24***	(0.21; 0.28)
**Socio-demographic factors**				
Country of birth (*Australian born*)				
Other English speaking country	−0.03	(−0.28; 0.21)	−0.05	(−0.26; 0.17)
Europe	0.33	(−0.15; 0.81)	−0.07	(−0.54; 0.40)
Asia	0.98***	(0.61; 1.34)	0.97***	(0.62; 1.31)
Other	0.20	(−0.30; 0.70)	0.07	(−0.39; 0.54)
Area of residence (*Urban*)				
Rural	−0.55***	(−0.64; −0.47)		
Remote	−0.61***	(−0.81; −0.41)		
Educational qualification (*University degree/higher degree*)				
Less than 12 years of school	−0.13	(−0.30; 0.04)	0.42***	(0.26; 0.58)
Completed 12 years of school	0.10	(−0.03; 0.23)	0.26***	(0.15; 0.38)
Post school/technical school	−0.01	(−.013; 0.11)	0.18***	(0.08; 0.28)
Marital status (*Single*)				
De facto	0.07	(−0.04; 0.17)	−0.19***	(−0.29; −0.09)
Married	−0.14**	(−0.25; −0.03)	−0.32***	(−0.41; −0.22)
Separated/divorced/widowed	0.08	(−0.12; 0.28)	−0.12	(−0.32; 0.08)
Number of children (*None*)				
1	−0.91***	(−1.02; −0.79)	−0.48***	(−0.59; −0.38)
2	−1.49***	(−1.62; −1.37)	−0.96***	(−1.08; −0.85)
≥3	−1.91***	(−2.07; −1.74)	−1.21***	(−1.36; −1.07)
**Work-related factors**				
Occupational status (*Professional*)				
No paid job	−0.43***	(−0.57; −0.29)	−0.15*	(−0.28; −0.02)
Blue collar	−1.30***	(−1.48; −1.13)	−0.06	(−0.21; 0.10)
White collar	0.25***	(0.15; 0.36)	−0.01	(−0.10; 0.08)
Hours worked per week (*35–40*)				
None	−0.64***	(−0.79; −0.49)	0.27***	(0.13; 0.40)
1-15	−0.77***	(−0.90; −0.63)	0.01	(−0.11; 0.13)
16-24	−0.69***	(−0.82; −0.55)	−0.07	(−0.19; 0.05)
25-34	−0.71***	(−0.85; −0.58)	−0.06	(−0.18; 0.06)
41-48	0.13*	(0.03; 0.24)	−0.06	(−0.15; 0.03)
≥49	0.10	(−0.03; 0.24)	−0.08	(−0.19; 0.04)
**Lifestyle factors**				
Being active	−0.28***	(−0.35; −0.21)	−0.23***	(−0.29; −0.17)
Smoking status (*Non smoker*)				
Ex-smoker			−0.01	(−0.10; 0.08)
Current smoker			0.13*	(0.03; 0.23)
Alcohol consumption (*Low risk drinker*)				
Non drinker			0.18**	(0.05; 0.30)
Rare drinker			0.15***	(0.08; 0.23)
Risky/high risk drinker			0.25**	(0.07; 0.44)
Being somewhat stressed	0.32***	(0.24; 0.39)	0.22***	(0.15; 0.28)

BMI, body mass index; CI, confidence interval; ^a^Women’s age at each survey (not only baseline age) was included in the model; ^b^Values for BMI signify 5 steps (i.e. 5 BMI-points) on the determinant scale; ^c^Educational qualification was retained in the model for week-day sitting with reference category ‘Completed 12 years of education’ with a significant outcome for ‘Less than 12 years of school’; -.0.24 (−0.40;-0.07); *p* < .01; *p < .05; **p < .01; ***p < 001.

Significant associations with *higher* sitting time were found for BMI, country of birth, education, occupation, hours worked, smoking, alcohol and stress. On *both* week- and weekend-days, women born in Asia, and those who reported being somewhat stressed sat more than Australian born and non-stressed women, respectively. There was also a positive association between BMI and sitting time on both week- and weekend-days. On week-days, women in white collar occupations and those working 41–48 hours per week sat more than professional women and usual full-time (35–40 hours) working women, respectively. On weekend-days, those with less than University level education and those who reported no paid work sat more than women with a degree, and full-time workers, respectively. Finally, current smokers and non-drinkers, rare and risky/high risk drinkers had higher sitting time on weekend-days than non-smokers and low risk drinkers.

### Multivariable associations with lower sitting time

There were also significant associations between area of residence, education, marital status, number of children, occupation, hours worked and physical activity with *lower* sitting time. On both week- and weekend-days, married women, those with any children, and those categorized as active sat less than single, childless and inactive women, respectively. Also, on both week- and weekend-days women without a paid job sat less than professional working women. On week-days, women living in rural and remote areas, those with less than 12 years of education, blue collar workers and women working less than 34 hours a week sat significantly less than women in the respective referent categories. Like their married counterparts, defacto women sat less than single women, but only on weekend-days.

### Robustness of the associations

When the univariable and multivariable analyses were repeated for women who completed all four surveys, (see Additional file 1 and Additional file 2) some of the associations between the explanatory factors and sitting time were slightly attenuated, but most remained significant. For week-day sitting the most important differences were that working long hours (41–48 hours a week) was no longer significantly associated with sitting time and that both smoking and alcohol consumption were included in the week-day model, with current smokers and non-drinking women sitting less than non-smoking women and low risk drinkers, respectively. For weekend-day sitting, the most important differences were that smoking was excluded from the model, and being a risky/high risk drinker was no longer significantly associated with sitting time.

## Discussion

This prospective study showed that young adult women sit around 6 hours a day on average on a week-day and around 5 hours a day on average on a weekend-day. Furthermore, sitting time declined slightly in young adult women over the nine years follow up, as they moved from their early twenties into their early thirties. The results provide insight into the complex biological, socio-demographic, work-related and lifestyle determinants of sitting time, some of which are associated with higher, and others with lower sitting time. Some of these determinants are similar to those reported by Rhodes and colleagues [8] - whose review focussed largely on cross-sectional studies of TV time. Our results however, improve understanding of the determinants of sitting time in young adult women, based on longitudinal assessment of the determinants of both week-day and weekend-day sitting. The findings show that sitting time changes as work and family responsibilities develop during this life stage.

### Body mass index (biological factor)

Our results showed that women with higher BMI sit more on both week- and weekend-days. Existing literature suggests an ambiguous relationship between BMI and sedentary behaviour. For example, based on a review of longitudinal studies, Proper and colleagues [4] concluded insufficient evidence for a relationship between sedentary behaviour and body weight/BMI gain or overweight and obesity. Yet, Rhodes and colleagues [8] found some evidence in their review of a positive relationship between certain types of sedentary behaviour (i.e., TV and general screen viewing) and BMI. The majority of studies included in both reviews examined whether more/less sitting time was associated with favorable/unfavorable body composition markers; i.e., they used sedentary behaviour as the independent variable. In our study, sedentary behaviour was the dependent variable, and it showed that women with higher BMI sit more on both week- and weekend-days. It may well be that women experience detrimental physical and emotional consequences of having higher BMI [20], which may cause them to sit more. This is in line with the finding from the mid-age ALSWH cohort, which raised the issue of whether sitting causes weight gain or higher weight causes more sitting [21].

### Country of birth, area of residence, educational qualification, marital status, and number of children (socio-demographic factors)

Our findings suggest that women born in Asia spend more time sitting on both week- and weekend-days than Australian born women. A systematic mixed-methods review on activity levels of South Asian women [22] has reported that only two recent studies-both performed in the UK-, have examined sedentary time (e.g., not the absence of physical activity) in this population. However, one of these studies did not report results on sitting time separately for women [23], and the other study made no comparison between Asian immigrants and ‘natives’ [24]. Future research on sedentary behaviour with female ethnically diverse groups is needed, as some groups appear to be at higher risk of having an inactive lifestyle, with both lower levels of physical activity and higher levels of sitting time. These populations should be a priority focus when developing interventions that discourage sitting.

Regarding education, we found evidence for higher sitting time among women with low education. Interestingly, our models showed that low education was negatively related with week-day sitting time, but positively related with weekend-day sitting. This points towards the likelihood of lower and technical educated women being in jobs where they are ‘on their feet’ all day on week-days. Most studies included in the Rhodes et al. review [8] presented significant associations between lower values of formal education and higher levels of TV viewing. However, no association with general sitting time was found. Our findings suggest that the well-documented associations between socio-economic status (including education) and unhealthy behaviours may apply to sitting time, *but only on weekends*. Pampel and colleagues [25] offer a comprehensive overview of the mechanisms/explanations that may underlie the relationship between low SES (including low education) and unhealthy behaviours, among which are higher rates of deprivation and stress, lack of knowledge and access to information about health risks, less efficacy and agency, and fewer financial aids. Our findings, and the research discussed above, suggest that low educated women should be targeted for intervention efforts that promote an active lifestyle, especially during the weekend.

The results of the multilevel analyses showed that married women sat significantly less on both week- and weekend-days than single women. Also, women in a de facto relationship (living together but not legally married) sat less than single women, but only on weekend-days. Vernon et al. [26] studied an American sample of 23,625 women (aged 22–65) and found that married women participated less in leisure activities like television watching, computer use, relaxing, and phone conversations, but spent more time doing household activities such as food preparation, housework and primary childcare, than single women. Furthermore, we found that having any number of children, compared with being childless, was associated with less sitting time on both week- and weekend-days. Likewise, Candelaria and colleagues [27] reported lower sitting time for mothers (and fathers) compared with nonparents. In line with our results, their study showed a direct inverse relationship between number of children and sitting time.

### Work-related factors

Hours worked per week and occupational status were consistently associated with week-day sitting. Overall, women in white collar occupations, professionals and women working full time or more, spent most time sitting during the week. Jans et al. [28] also found that, among 7720 Dutch workers, professionals (i.e., legislators and senior managers, scientific and artistic professions) and white collar workers (i.e., clerks) sat longer than the average Dutch worker (i.e., more than 7 hours a day). No distinction was made between men and women. Regarding hours worked per week, a cross-sectional study using accelerometry to assess sitting time in US women (aged 20–60) showed no significant difference between the part time, full time and non-employed [29], which is in contrast with our results. It does however seem logical that full-time working professional and white collar women should have higher sitting time during the week, as many of their jobs typically require sitting at a desk [30]. However, on weekend-days, the findings were more complex. Women who had no *paid* work (but may have worked in a voluntary capacity, or in a family business) sat less than full-time working women on weekend-days. This may reflect their work on a farm or in a shop that remained ‘open’ on weekends. In contrast, women with no work hours (paid or unpaid) sat more than full-time working women on weekends, possibly because they had more ‘free’ time for screen based activities.

### Lifestyle factors

Our results showed that inactive women spent more time sitting than their active counterparts. Although the evidence currently considers sedentary behaviour as a unique behaviour (rather than the absence of physical activity), physical activity and sitting seem to be interrelated in our population. In line with the idea that adverse health behaviours tend to cluster within individuals [31], we also found that stressed women spent more time sitting on both week- and weekend-days, and that current smokers sat more than non-smokers on weekend-days. The univariable models showed that on week-days, smoking was significantly associated with less sitting time, which could be explained by the fact that those in work have to leave their desk/home to smoke outside. The fact that this relationship did not persist in the multivariable model (which showed smokers sitting more on weekend-days), points to the complex relationships between smoking and education, profession and parenting roles. Relationships with alcohol were also complex, but could be interpreted to mean that high risk drinkers sit more at weekends, possibly reflecting their ‘pub/club’ culture, while higher weekend sitting among non/rare drinkers may reflect a propensity for more sedentary leisure activities, such as reading and crafts.

Overall, despite the claim that physical activity and sitting time are distinct behaviours [32] our results showed that, in this population based sample of young adult women, the determinants of sitting are remarkably similar to those for physical activity (e.g., higher BMI, being born in Asia, low education, being married, having children, smoking, and stress) [Uijtdewilligen L, Peeters GEE, van Uffelen JGZ, Twisk JWR, Singh AS, Brown WJ; unpublished observations]. However, it is important to note that the associations between the determinants and the two behaviours are not always in the opposite direction. For example, married women and women with children, tend to participate in less physical activity [33], but they also spend less time sitting. This is because mothers of young children spend more time in low intensity physical activities, such as household chores and child care [34]. On the whole, it is concluded that women with higher BMI, those of Asian descent, those with low education, and women who are somewhat stressed, are at greater risk of *both* sitting more (on week-days and weekend-days) and participating less in physical activity [Uijtdewilligen L, Peeters GEE, van Uffelen JGZ, Twisk JWR, Singh AS, Brown WJ; unpublished observations]. This information could be important when selecting target groups for interventions that aim to both increase physical activity and reduce sitting time.

### Strengths and limitations

The main strengths of our study are the large population based sample and the collection of data over a period of nine years when the women were young adults; a time when socio-demographic, work-related and lifestyle factors change frequently. Another strength is the statistics applied to assess the relationship between biological, socio-demographic, work-related and lifestyle factors and sitting time that capture the changing status of all the determinants and sitting time, as well as the relationships between them, over time. The main limitation of the study is the use of a self-report measure to assess sitting time. Use of objective methods was not feasible in this a sample due to financial and logistic constraints. Finally, as our sample only included young adult women, the results may not be generalisable to men, or to mid-age or older women.

## Conclusion

As evidence on the adverse effects of sitting accumulates, it is important to develop strategies to discourage this behaviour. This study showed that many young women spend a substantial proportion of their waking hours sitting. Women with higher BMI, women born in Asia, those with higher level occupations and long working hours, were most at risk of higher sitting time. These results can be used for the identification of at-risk groups and improving intervention development.

## Competing interest

The authors declare that they have no competing interests.

## Authors' contributions

LU contributed to the study concept and design, performed the statistical analyses, interpreted the data, and drafted the manuscript. JT contributed to the data analyses and interpretation, and critically revised the manuscript for important intellectual content. MJ, WvM and AS contributed to the study design, and critically reviewed the manuscript. WB was involved in the conceptualization, design, management and data collection for the ALSWH study; she contributed to the study concept and design, advised on analyses, and revised the manuscript for important intellectual content. All authors read and approved the final manuscript and participated sufficiently in the work to take public responsibility for appropriate portions of the content.

## Supplementary Material

Additional file 1 Results of age-adjusted univariable GEE analyses of women completing all four surveys. Click here for file

Additional file 2 Results of age-adjusted multivariable GEE analyses of women completing all four surveys. Click here for file
