# Supplementary material for: Biological, socio-demographic, work and lifestyle determinants of sitting in young adult women: a prospective cohort study
Source: Int J Behav Nutr Phys Act. 2014 Jan 24;11:7. doi: 10.1186/1479-5868-11-7 (PMC3904408; doi:10.1186/1479-5868-11-7)
Supplement: Additional file 1 — Results of age-adjusted univariable GEE analyses of women completing all four surveys. [file 1479-5868-11-7-S1.doc]

**Additional file 1**. Results of age-adjusted^a^ univariable GEE analyses of women completing all four surveys (n=5224) presenting associations of biological, socio-demographic, work-related and lifestyle factors with sitting time by each category of the explanatory variable compared with the reference group (in parentheses)

|  | **Week-day sitting (hours/day)** | | | **Weekend-day sitting (hours/day)** | | |
| --- | --- | --- | --- | --- | --- | --- |
| Explanatory variable *(reference)* | B (95% CI) | | | B (95% CI) | | |
| **Socio-demographic factors** |  |  |  |  |  |  |
| BMI^b^ | 0.16*** | (0.11; | 0.22) | 0.29*** | (0.24; | 0.34) |
| Country of birth *(Australian born)* |  |  |  |  |  |  |
| Other English speaking country | 0.01 | (-0.33; | 0.35) | -0.09 | (-0.36; | 0.17) |
| Europe | 0.52 | (-0.17; | 1.22) | -0.33 | (-0.92; | 0.25) |
| Asia | 1.92*** | (1.36; | 2.48) | 1.04*** | (0.51; | 1.56) |
| Other | 0.15 | (-0.72; | 1.02) | 0.19 | (-0.45; | 0.83) |
| Area of residence *(Urban)* |  |  |  |  |  |  |
| Rural | -0.89*** | (-1.00; | -0.78) | -0.09 | (-0.18; | 0.00) |
| Remote | -0.92*** | (-1.18; | -0.66) | 0.05 | (-0.16; | 0.26) |
| Educational qualification *(University degree/ higher degree)* |  |  |  |  |  |  |
| Less than 12 years of school | -0.92*** | (-1.14; | -0.70) | 0.22* | (0.04; | 0.41) |
| Completed 12 years of school | -0.33*** | (-0.49; | -0.17) | 0.19** | (0.06; | 0.32) |
| Post school/ technical school | -0.33*** | (-0.48; | -0.19) | 0.16** | (0.04; | 0.27) |
| Marital status *(Single)* |  |  |  |  |  |  |
| De facto | 0.02 | (-0.11; | 0.15) | -0.16** | (-0.27; | -0.04) |
| Married | -0.69*** | (-0.82; | -0.57) | -0.45*** | (-0.56; | -0.34) |
| Separated/ divorced/ widowed | -0.29* | (-0.55; | -0.03) | -0.20 | (-0.46; | 0.05) |
| Number of children *(None)* |  |  |  |  |  |  |
| 1 | -1.41*** | (-1.52; | -1.29) | -0.32*** | (-0.43; | -0.22) |
| 2 | -2.21*** | (-2.34; | -2.07) | -0.88*** | (-0.99; | -0.77) |
| ≥3 | -2.76*** | (-2.94; | -2.57) | -1.00*** | (-1.16; | -0.85) |
| **Work-related factors** |  |  |  |  |  |  |
| Occupational status *(Professional)* |  |  |  |  |  |  |
| No job | -1.40*** | (-1.53; | -1.27) | -0.19*** | (-0.29; | -0.08 |
| Blue collar | -1.60*** | (-1.81; | -1.40) | 0.00 | (-0.18; | 0.19 |
| White collar | 0.07 | (-0.05; | 0.20) | 0.02 | (-0.08; | 0.12 |
| Hours worked per week *(35-40)* |  |  |  |  |  |  |
| None | -1.55*** | (-1.68; | -1.41) | -0.19** | (-0.30; | -0.07) |
| 1-15 | -1.43*** | (-1.58; | -1.28) | -0.31*** | (-0.43; | -0.18) |
| 16-24 | -1.22*** | (-1.37; | -1.07) | -0.35*** | (-0.48; | -0.22) |
| 25-34 | -0.90*** | (-1.06; | -0.75) | -0.17* | (-0.31; | -0.04) |
| 41-48 | 0.09 | (-0.03; | 0.21) | -0.04 | (-0.14; | 0.07) |
| ≥49 | 0.04 | (-0.12; | 0.20) | -0.03 | (-0.16; | 0.10) |
| **Lifestyle factors** |  |  |  |  |  |  |
| Being inactive | -0.10* | (-0.18; | -0.01) | -0.13*** | (-0.21; | -0.06) |
| Smoking status *(Non smoker)* |  |  |  |  |  |  |
| Ex-smoker | -0.16* | (-0.30; | -0.02) | 0.05 | (-0.06; | 0.17) |
| Current smoker | -0.11 | (-0.26; | 0.04) | 0.19** | (0.06; | 0.32) |
|  |  |  |  |  |  |  |
|  |  |  |  |  |  |  |
|  |  |  |  |  |  |  |
|  |  |  |  |  |  |  |
| Alcohol consumption *(Low risk drinker)* |  |  |  |  |  |  |
| Non drinker | -0.49*** | (-0.66; | -0.32) | 0.19* | (0.05; | 0.34) |
| Rare drinker | -0.25*** | (-0.36; | -0.14) | 0.15** | (0.06; | 0.25) |
| Risky/ high risk drinker | 0.28* | (0.04; | 0.53) | 0.24* | (0.01; | 0.46) |
| Being somewhat stressed | 0.28*** | (0.19; | 0.36) | 0.18*** | (0.11; | 0.26) |

BMI, body mass index; CI, confidence interval;

^a^ Women’s age at each survey (not only baseline age) was included in the model;

^b^ Values for BMI signify 5 steps (i.e. 5 BMI-points) on the determinant scale;

**p*<.05;

***p*<.01;

****p*<.001.
