# Supplementary material for: Biological, socio-demographic, work and lifestyle determinants of sitting in young adult women: a prospective cohort study
Source: Int J Behav Nutr Phys Act. 2014 Jan 24;11:7. doi: 10.1186/1479-5868-11-7 (PMC3904408; doi:10.1186/1479-5868-11-7)
Supplement: Additional file 2 — Results of age-adjusted multivariable GEE analyses of women completing all four surveys. [file 1479-5868-11-7-S2.doc]

**Additional file 2.** Results of age-adjusted^a^ multivariable GEE analyses of women completing all four surveys (n=5224) presenting associations of biological, socio-demographic, work-related and lifestyle factors with sitting time by each category of the explanatory variable compared with the reference group (in parentheses)

BMI, body mass index; CI, confidence interval;

|  | **Week-day sitting (hours/day)** | | | **Weekend-day sitting (hours/day)** | | |
| --- | --- | --- | --- | --- | --- | --- |
| Explanatory variable *(reference)* | B (95% CI) | | | B (95% CI) | | |
| **Socio-demographic factors** |  |  |  |  |  |  |
| BMI^b^ | 0.21*** | (0.16; | 0.27) | 0.27*** | (0.22; | 0.32) |
| Country of birth *(Australian born)* |  |  |  |  |  |  |
| Other English speaking country | -0.18 | (-0.49; | 0.14) | -0.14 | -0.40; | 0.12) |
| Europe | 0.32 | (-0.29; | 0.94) | -0.27*** | (-0.88; | 0.34) |
| Asia | 1.40*** | (0.86; | 1.93) | 1.05 | (0.53; | 1.57) |
| Other | 0.04 | (-0.78; | 0.85) | 0.31 | (-0.36; | 0.98) |
| Area of residence *(Urban)* |  |  |  |  |  |  |
| Rural | -0.61*** | (-0.72; | -0.50) |  |  |  |
| Remote | -0.65*** | (-0.91; | -0.39) |  |  |  |
| Educational qualification *(University degree/ higher degree)* |  |  |  |  |  |  |
| Less than 12 years of school | 0.09 | (-0.15; | 0.32) | 0.44*** | (0.23; | 0.65) |
| Completed 12 years of school | 0.28** | (0.11; | 0.45) | 0.35*** | (0.20; | 0.50) |
| Post school/ technical school | 0.14 | (-0.02; | 0.29) | 0.21** | (0.08; | 0.34) |
| Marital status *(Single)^c^* |  |  |  |  |  |  |
| De facto | 0.08 | (-0.05; | 0.22) | -0.14* | (-0.26; | -0.02) |
| Married | -0.11 | (-0.25; | 0.02) | -0.27*** | (-0.39; | -0.16) |
| Separated/ divorced/ widowed | 0.09 | (-0.25; | 0.02) | -0.12 | (-0.38; | 0.15) |
| Number of children *(None)* |  |  |  |  |  |  |
| 1 | -0.84*** | (-0.99; | -0.70) | -0.42*** | (-0.55; | -0.28) |
| 2 | -1.57*** | (-1.73; | -1.41) | -0.99*** | (-1.13; | -0.85) |
| ≥3 | -2.02*** | (-2.23; | -1.80) | -1.20*** | (-1.38; | -1.01) |
| **Work-related factors** |  |  |  |  |  |  |
| Occupational status *(Professional)* |  |  |  |  |  |  |
| No job | -0.40*** | (-0.57; | -0.22) | -0.16* | (-0.32; | 0.00) |
| Blue collar | -1.35*** | (-1.57; | -1.12) | -0.13 | (-0.34; | 0.07) |
| White collar | 0.29*** | (0.16; | 0.43) | -0.07 | (-0.18; | 0.05) |
| Hours worked per week *(35-40)* |  |  |  |  |  |  |
| None | -0.66*** | (-0.85; | -0.47) | 0.23** | (0.07; | 0.40) |
| 1-15 | -0.81*** | (-0.98; | -0.64) | 0.01 | (-0.13; | 0.16) |
| 16-24 | -0.72*** | (-0.89; | -0.56) | -0.08 | (-0.23; | 0.07) |
| 25-34 | -0.70*** | (-0.86; | -0.53) | -0.02 | (-0.16; | 0.13) |
| 41-48 | 0.06 | (-0.07; | 0.18) | -0.07 | (-0.18; | 0.04) |
| ≥49 | 0.05 | (-0.12; | 0.21) | -0.08 | (-0.22; | 0.06) |
| **Lifestyle factors** |  |  |  |  |  |  |
| Being inactive | -0.29*** | (-0.37; | -0.20) | -0.17*** | (0.04; | 0.24) |
| Smoking status *(Non smoker)* |  |  |  |  |  |  |
| Ex-smoker | -0.09** | (-0.23; | 0.05) |  |  |  |
| Current smoker | -0.22 | (-0.37; | -0.06) |  |  |  |
|  |  |  |  |  |  |  |
|  |  |  |  |  |  |  |
|  |  |  |  |  |  |  |
|  |  |  |  |  |  |  |
| Alcohol consumption *(Low risk drinker)* |  |  |  |  |  |  |
| Non drinker | -0.18* | (-0.36; | -0.01) | 0.19* | (0.04; | 0.34) |
| Rare drinker | -0.08 | (-0.20; | 0.03) | 0.14** | (0.04; | 0.24) |
| Risky/ high risk drinker | 0.16 | (-0.08; | 0.40) | 0.14 | (-0.10; | 0.38) |
| Being somewhat stressed | 0.28*** | (0.19; | 0.37) | 0.16*** | (0.08; | 0.24) |

^a^ Women’s age at each survey (not only baseline age) was included in the model;

^b^ Values for BMI signify 5 steps (i.e. 5 BMI-points) on the determinant scale;

^c^ Marital status was retained in the model for week-day sitting with reference category ‘Married’ with a significant outcome for ‘Single’; .0.20 (0.07; 0.33); *p*<.01;

**p*<.05;

***p*<.01;

****p*<001.
